# Blood–Brain Barrier Disruption in Neuroimmunological Disease

**DOI:** 10.3390/ijms251910625

**Published:** 2024-10-02

**Authors:** Fumitaka Shimizu, Masayuki Nakamori

**Affiliations:** Department of Neurology and Clinical Neuroscience, Yamaguchi University Graduate School of Medicine, Ube 755-8505, Japan; mnakamor@yamaguchi-u.ac.jp

**Keywords:** blood–brain barrier, neuroimmunological disease, multiple sclerosis, neuromyelitis optica spectrum disorder, autoimmune encephalitis, paraneoplastic neurological syndrome

## Abstract

The blood–brain barrier (BBB) acts as a structural and functional barrier for brain homeostasis. This review highlights the pathological contribution of BBB dysfunction to neuroimmunological diseases, including multiple sclerosis (MS), neuromyelitis optica spectrum disorder (NMOSD), myelin oligodendrocyte glycoprotein antibody-associated disease (MOGAD), autoimmune encephalitis (AE), and paraneoplastic neurological syndrome (PNS). The transmigration of massive lymphocytes across the BBB caused by the activation of cell adhesion molecules is involved in the early phase of MS, and dysfunction of the cortical BBB is associated with the atrophy of gray matter in the late phase of MS. At the onset of NMOSD, increased permeability of the BBB causes the entry of circulating AQP4 autoantibodies into the central nervous system (CNS). Recent reports have shown the importance of glucose-regulated protein (GRP) autoantibodies as BBB-reactive autoantibodies in NMOSD, which induce antibody-mediated BBB dysfunction. BBB breakdown has also been observed in MOGAD, NPSLE, and AE with anti-NMDAR antibodies. Our recent report demonstrated the presence of GRP78 autoantibodies in patients with MOGAD and the molecular mechanism responsible for GRP78 autoantibody-mediated BBB impairment. Disruption of the BBB may explain the symptoms in the brain and cerebellum in the development of PNS, as it induces the entry of pathogenic autoantibodies or lymphocytes into the CNS through autoimmunity against tumors in the periphery. GRP78 autoantibodies were detected in paraneoplastic cerebellar degeneration and Lambert–Eaton myasthenic syndrome, and they were associated with cerebellar ataxia with anti-P/Q type voltage-gated calcium channel antibodies. This review reports that therapies affecting the BBB that are currently available for disease-modifying therapies for neuroimmunological diseases have the potential to prevent BBB damage.

## 1. Introduction

**Figure 1:**
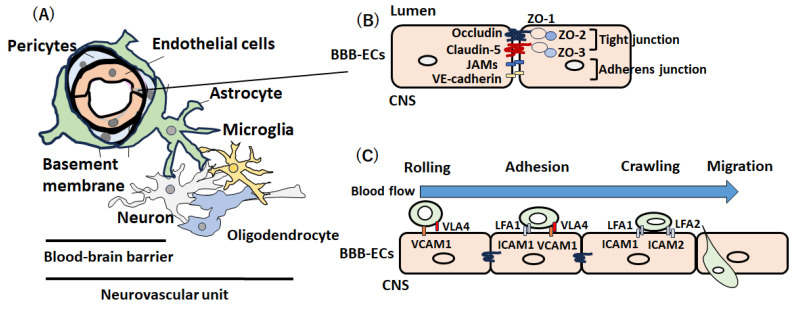
**Structure of the blood–brain barrier (BBB)**. (**A**) The blood–brain barrier (BBB) consists of endothelial cells, pericytes, astrocytes, and the basement membrane. The neurovascular unit (NVU) is composed of endothelial cells, pericytes, and astrocytes of the BBB and neurons, oligodendrocytes, and microglia, which closely communicate with each other in order to regulate brain homeostasis. (**B**) Tight junctions (claudin-5, occludin, ZO-1, ZO-2, and ZO-3) and adhesion junctions (JAMs and VE-cadherin) between BBB endothelial cells (BBB-ECs) form the BBB. (**C**) Transcellular migration of lymphocytes involves the following 4 steps: (1) in the rolling process, activated lymphocytes slow their flow speed due to the interaction of VLA-4 from the surface of lymphocytes with vascular cell adhesion molecules 1 (VCAM-1) on BBB-ECs; (2) in adhesion pathways, the lymphocytes adhere to endothelial cells and transverse the BBB by coupling the VLA-4 and LFA-1 expressed on lymphocytes with the endothelial cell receptor (VCAM-1 and intracellular adhesion molecules (ICAM-1); (3) during adhesion, interaction between VCAM-1 and ICAM on BBB-ECs and their ligands (LFA-1 and VLA-4) on leukocytes induces the arrest of immune cells from the blood by the brain endothelial cells; and (4) interaction between ICAM-1 and ICAM-2 and their ligands (LFA-1 and LFA-2) is involved in crawling and migration.

The blood–brain barrier (BBB) plays an important role in protecting the central nervous system (CNS) from potentially harmful circulating pathogens [1,2]. The BBB consists of brain microvascular endothelial cells (BMECs) surrounded by pericytes and astrocytes and ensheathed in two basement membranes (the vascular basement membrane and the glia limitans) [1,2]. These cells, in addition to neurons and perivascular microglia, constitute the neurovascular unit (NVU) [3,4] (Figure 1A). BMECs form a physical barrier through tight junctions and adherens junctions to prevent the entry of blood cells and other molecules, and they maintain brain homeostasis by controlling nutrient, water, and molecule exchanges and removing waste products from the CNS through transporters [5]. Tight junction proteins include claudins (especially claudin-5) and occludin, which have intracellular domains that interact with ZO-1. At the intracellular level, ZO-1, ZO-2, and ZO-3 establish a link between transmembrane proteins and the actin cytoskeleton to maintain cytoskeletal integrity [6,7] (Figure 1B). Adherens junctions are composed of VE-cadherin and catenins [6,7].

The major physiological roles of the intact BBB are (1) the restriction of plasma macromolecules into the brain, (2) the maintenance of ionic metastasis, (3) the uptake of brain nutrients, (4) the regulation of optimal levels of neurotransmitters, (5) the protection of the brain against neurotoxins, and (6) the elimination of substances from the brain [8,9].

The breakdown of the BBB gives rise to increased paracellular permeability of humoral factors through the decrease in tight junctions and increased inflammatory cell trafficking across the BBB via the upregulation of adhesion molecules [1,9]. Pathological autoantibodies that target molecules on the BBB’s endothelial cells or several inflammatory cytokines, such as TNF-α and IFN-γ, can activate BBB endothelial cells by upregulating proinflammatory signals, such as NF-κB, resulting in a decrease in tight junctions and an increase in adhesion molecules [2,10].

The breakdown of the BBB is associated with several neuroimmunological diseases, including multiple sclerosis (MS), neuromyelitis optica (NMO), myelin oligodendrocyte glycoprotein antibody-associated disease (MOGAD), autoimmune encephalitis (AE), and neuropsychiatric systemic lupus erythematosus (NPSLE) [2,10]. Whether or not the breakdown of the BBB is a causative factor in these neuroimmunological diseases remains unclear, but recent data suggest that BBB alteration may be the cause of the development of the disease in NMO, MOGAD, and MS during an acute attack, and BBB dysfunction may be a consequence of progressive MS [1,2,9].

This review discusses the significance and molecular mechanisms of BBB disruption in neuroimmunological diseases.

## 2. Multiple Sclerosis

### 2.1. The BBB Breakdown in Multiple Sclerosis

Multiple sclerosis is the most common chronic inflammatory demyelinating disease affecting the CNS [11,12]. It affects the brain, spinal cord, and optic nerves. Relapsing–remitting MS (RRMS) is characterized by intermittent neurological disturbance (relapse) followed by complete or incomplete recovery [13]. Throughout the disease course, MS usually begins as RRMS, and 30–60% of RRMS patients shift to a phase of secondary progressive MS, characterized by gradual clinical worsening without relapse [14,15,16,17]. Multiple genetic polymorphisms with environmental and endogenous triggers are believed to lead to the formation of demyelinating plaques with inflammation and, ultimately, neurodegeneration [18]. Neurodegenerative processes, including axonal loss and gray matter atrophy, are major causes of neurological disability in secondary progressive MS (SPMS) [18,19].

In the early stages of MS, inflammatory BBB malfunction is associated with pathogenic immune cell infiltration, including T cells and B cells, immunoglobulin G, and inflammatory cytokines into the CNS parenchyma, although a normal BBB restricts the entry of immune cells into the CNS [20,21,22]. Clinical findings show that newly formed lesions within the CNS can be detected through gadolinium (Gd) enhancement of the brain on T1-weighted magnetic resonance imaging (MRI) during relapse in MS [23]. This change is considered a feature of BBB disruption [23]. An increase in the cerebrospinal fluid (CSF)/serum albumin ratio in MS patients also reflects the movement of albumin from the blood to the CSF via BBB disruption [24].

In MS, the disruption of the BBB was considered to be transient, as the contrast effect of Gd enhancement on T1-weighted MRI did not last long. In histopathological findings, acute MS lesions demonstrated disruption of the BBB, supported by post-mortem evidence of focal microvascular leakage of albumin and the accumulation of inflammatory cells around the vessels [23]. Furthermore, abnormalities of the BBB, including vascular leakage and the decreased expression of tight junction proteins, were observed in both active and inactive lesions, as well as in normal-appearing white matter (NAWM) in RRMS and SPMS patients, suggesting that the persistent loss of BBB integrity may be involved in pathogenesis in both disease onset and progression [25].

### 2.2. Molecular Basis of BBB Disruption in RRMS

**Figure 2:**
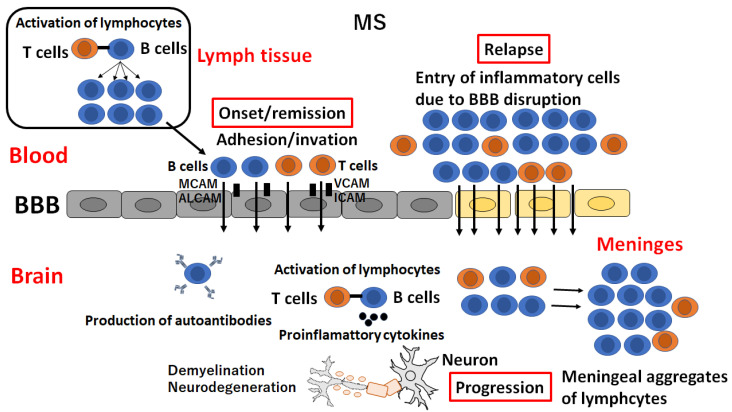
**Flow of lymphocytes in multiple sclerosis (MS).** After T and B lymphocytes are activated in the periphery, they infiltrate the CNS and trigger the central autoimmune response, leading to myelin and axonal damage during onset or remission in MS. The cell adhesion molecules (CAMs) expressed on the surface of inflamed endothelial cells, including vascular cell adhesion molecule 1 (VCAM-1), intracellular adhesion molecules (ICAM-1), melanoma cell adhesion molecules (MCAMs), and activated leukocyte cell adhesion molecules (ALCAMs), play a role in trans-endothelial immune cell infiltration. After activated lymphocytes disrupt the BBB, massive numbers of lymphocytes enter the CNS, leading to relapse in MS. Lymphocytes in the CNS aggregate in the meninges in progressive MS.

After T and B lymphocytes are activated in the periphery as a first step, they infiltrate the CNS and trigger a central autoimmune response, leading to myelin and axonal damage [26]. Both BBB disruption and leukocyte trafficking are the most important pathological processes in the active lesion (“the acute demyelinating brain lesion”) as well as the inactive lesion (“NAWM”) [27]. Leukocyte-derived proinflammatory cytokines activate endothelial cells and upregulate the expression of additional adhesion molecules, thus mediating the self-sustained CNS infiltration of more immune cells (Figure 2) [26,27]. During tethering, peripheral lymphocytes express the P-selectin glycoprotein ligand-1 (PSGL-1), which interacts with the ligand molecules expressed on endothelial cells (E- and P-selectins) and facilitates the capture of lymphocytes [28].

During the rolling process, endothelial cells express several chemokines, including CCL21 and CCL19, which activate the G protein-coupled receptor (GPCR) on the surface of the lymphocyte and stimulate the expression of integrin α4β1 (very late antigen-4: VLA-4) and lymphocyte function-associated antigen 1 (S) [29]. Activated lymphocytes slow their flow speed due to the interaction of VLA-4 and LFA-1 from the surface of lymphocytes with adhesion molecules from the surface of inflamed endothelial cells, including vascular cell adhesion molecule 1 (VCAM-1) and intracellular adhesion molecules (ICAM-1) [30]. In adhesion and transcellular pathways, lymphocytes adhere to endothelial cells and transverse the BBB by coupling VLA-4 and LFA-1 expressed on lymphocytes with endothelial cell receptors (VCAM-1 and ICAM-1) [30]. The interaction between VCAM-1 and ICAM and their ligands on leukocytes induces the arrest of immune cells from the blood in brain endothelial cells [31] (Figure 1C). Importantly, the upregulation of VCAM-1 was observed in the BBB endothelial cells around the active or inactive lesion or NAWM in autopsy cases of MS, suggesting that the activation of endothelial cells and the upregulation of VCAM-1 precede the formation of demyelination [32].

Natalizumab, a monoclonal antibody against α4β1 integrin, the ligand of VCAM-1, directly interferes with the transmigration of T and B lymphocytes across BBB endothelial cells [33]. In addition, other cell adhesion molecules (CAMs), including melanoma cell adhesion molecules (MCAMs), activated leukocyte cell adhesion molecules (ALCAMs), platelet and endothelial cell adhesion molecules (PECAMs), and dual immunoglobulin domain-containing cell adhesion molecules (DICAMs), play a role in trans-endothelial immune cell infiltration [34,35,36,37]. The upregulation of MCAM in brain endothelial cells recruits pathogenic Th1 and Th17 CD4+ T lymphocytes expressing MCAM from circulation during neuroinflammation in experimental autoimmune encephalomyelitis (EAE) and autopsied brain samples in MS [34]. The upregulation of ALCAMs in brain endothelial cells drives the entry of proinflammatory B lymphocytes expressing ALCAMs into the brain lesion in EAE and MS [35]. An increase in DICAM-expressing Th17 CD4+ T cells and the upregulation of the DICAM ligand on the brain endothelial cells upon inflammation and in MS lesions have been observed, and monoclonal antibodies against DICAM have been shown to reduce Th17 cell trafficking across the blood–brain barrier both in vitro and in vivo and to ameliorate both relapsing and progressive EAE [37]. Therefore, CAMs and their interacting ligands are attractive targets for novel therapies for RRMS.

### 2.3. BBB in Progressive MS

In the late stage of MS, cortical gray matter atrophy is correlated with cognitive decline and gait disturbances [38]. A total of 45% of RRMS patients and 75% of SPMS patients show effects on their daily working memory and verbal fluency tasks [38]. Gd-enhanced lesions on MRI, reflecting disruption of the BBB, were rarely observed in progressive MS, although fibrin deposition and tight junction abnormalities were found in the cortex in progressive MS in both active and inactive lesions and NAWM [25], suggesting persistent dysfunction of cortical BBB integrity in progressive MS [26]. We demonstrated that anti-galectin-3 autoantibodies in SPMS mediate the breakdown of the BBB through the degradation of claudin-5 and the upregulation of ICAM-1, and we reported that anti-galectin-3 antibodies were associated with persistent damage to the BBB [39]. Galectin-3 is a β-galactoside-binding lectin expressed both extra- and intracellularly in several cell types, and the activation of intracellular galectin-3 can induce the activation of the NFκB pathway [40,41]. Anti-galectin 3 antibodies also may prevent remyelination, causing morphological and functional differentiation of oligodendrocyte progenitor cells [42].

Gray matter atrophy can induce cortical hypoperfusion in progressive MS. Functional MRI reportedly shows that cerebral vascular reactivity, which is the change in cerebral blood flow upon stimulation with vasoactive compounds, is reduced in the gray matter of patients with MS [43]. This change was shown to be correlated with gray matter atrophy and lesion volume in patients with MS [43]. The disturbance of cerebral vascular reactivity reflects the dysfunction of the neurovascular coupling (NVC), which is linked to neurodegeneration. The vascular pathology hypothesis in MS states that vascular changes play a central role in MS pathogenesis [44]. Whether vascular pathology is a cause or consequence of neurodegeneration associated with cognitive impairment in MS remains elusive [45].

### 2.4. Fluid Biomarkers for BBB Disturbance in MS

Serum molecules associated with CNS cell damage, including neurofilament light chain (neuron), GFAP, and S100B (astrocytes), have been detected in MS and NMO using ultrasensitive single-molecular arrays, reflecting CSF drainage towards the peripheral compartment through a disrupted BBB. Neurofilament light chain concentrations in the blood and CSF are increased in newly diagnosed MS patients and correlate with relapse, new lesions on MRI, disease severity, and prognosis in MS [46]. An increase in the serum neurofilament light chain was shown to be related to the elevation of the CSF/serum quotient of albumin (Q Alb) and CSF-located CD80+ B cells and the presence of Gd-enhancement lesions on MRI, suggesting increased BBB permeability in MS [47], and treatment with disease-modifying therapies was found to decrease serum neurofilament light chain levels [47,48,49]. The serum concentration of GFAP, reflecting astrocyte damage, was higher in patients with progressive MS during relapse than healthy controls [50], but it increased in response to CNS injury, including BBB breakdown after TBI and intracerebral hemorrhaging [51]. Serum S100B is related to permeable BBB and S100B from serum and CSF, and it is likely secreted from astrocytes or Schwann cells. Increases in S100B are observed in RRMS at diagnosis, and they are related to disease severity and progression in MS [47].

Chemokines play a role in the recruitment of leukocytes to inflamed CNS sites. An increase in some chemokines, including CXCL8 (which mediates the recruitment of neutrophils secreted by macrophages or endothelial cells), CXCL10/interferon gamma-induced protein (IP)-10 (which mediates the recruitment of T cells and macrophages secreted by monocytes and endothelial cells), and CXCL13 (which mediates the recruitment of B cells secreted by B cells), can be observed in the CSF of patients with MS compared to non-inflammatory controls [52].

Levels of the soluble form of CAMs secreted by endothelial cells, including VCAM-1 (serum), MCAM (CSF), and PECAM-1, are increased in MS [36,53,54]. PECAM-1 is a cell adhesion molecule that is highly enriched at the interendothelial junctions of vascular endothelial cells and mediates neutrophil diapedesis across the vascular wall [36]. The serum concentration of soluble PECAM-1 (sPECAM-1) correlates with active, gadolinium-enhancing lesions on brain MRI in MS [26]. In addition, serum soluble VCAM-1 (sVCAM-1) and sPECAM-1 are considered to be markers of BBB disruption [26,53]. sVCAM-1 is cut from the surface of BBB endothelial cells during inflammation, and it correlates with the presence of Gd-enhancing lesions on brain MRI and with the clinical disease activity in RRMS patients, but it remains low in progressive MS [53].

### 2.5. Possible Causes of BBB Disturbance in MS

Genetic and environmental factors associated with MS can contribute directly and indirectly to BBB disturbances. A genome-wide association study showed that more than 230 genetic variants in MS and human leukocyte antigen (HLA)-DRB1 polymorphisms were associated with MS risk [55]. The single-nucleotide polymorphism (SNP) of ALCAM (rs6437585) is associated with risk for and progression of MS, while the SNP of VCAM-1 (rs11581062) is a risk factor for MS [35,56]. VCAM-1 and ALCAM play an important role in immune cell adhesion and transmigration and are linked to BBB disturbance in the development of MS.

A decrease in the serum 25-hydroxyvitamin D level is associated with an increased risk of MS onset and disease progression [57]. In a clinical study, vitamin D3 with interferon-beta (IFN-β) reduced the number of new Gd-enhanced lesions in RRMS compared to the placebo with IFN-β, which suggests a role for vitamin D in repairing the BBB’s function [58]. The active form of vitamin D (1,25(OH)2D3) enhances the barrier function by upregulating claudin-5 and reducing VCAM-1 expression [59,60].

Infection with Epstein–Barr virus (EBV) is an important causal factor for increased risk of subsequent MS. The risk of MS was 32-fold higher following EBV infection, and the serum concentration of the neurofilament light chain was increased after EBV seroconversion in MS [61]. EBV can contribute to the development of MS through molecular mimicry between the chronic presentation of viral antigens as a potential source of autoreactivity and CNS proteins, such as anoctamin-2 and GlialCAM [62]. Autoantibodies against anoctamin-2 (an ion channel expressed in the CNS) or GlialCAM (a component of glial cells in the brain) can recognize the fragment of EBV nuclear antigen 1, and they were increased in MS patients [63,64]. Upregulation of ICAM-1 and CCL5 and increased adherence of leukocytes have been observed in BBB endothelial cells infected with EBV [65].

Smoking is a risk factor for the onset and progression of MS [66]. Nicotine can enhance BBB permeability by downregulating tight junction proteins [67]. In addition, concussion and brain trauma during adolescence are associated with the onset of MS [68]. Some reports have suggested that brain trauma temporarily increases BBB permeability [69].

### 2.6. Dysfunction of NVU in MS

The NVU is composed of endothelial cells, pericytes, astrocytes, neurons, microglia, and extracellular matrix components, which play a role in coupling cerebral blood flow with neural activity in different regions of the brain to regulate vasodilation and vasoconstriction, when needed [3,4]. Pericytes located between endothelial cells and astrocytes or neurons receive signals from neurons and function as regulators of BBB permeability, hemodynamic responses under neuroinflammation, and clearance of toxic metabolism [3]. In MS lesions, increased permeability of the NVU is mainly due to dysfunction of the BBB endothelial cells, which induce paracellular leakage and leukocyte migration across the BBB [1,27]. Perivascular astrogliosis and the retraction of astrocyte endfeet contribute to the dysfunction of pericytes and endothelial cells, which further exacerbate the increased permeability of the BBB and the poor hemodynamic response in MS [7,27]. Dysfunction of the NVU in progressive MS is associated with cerebral hypoperfusion [44,45]. Demyelinating MS lesions show oligodendrocyte degeneration, partly due to hypoxic injury. Hypoxia due to NVU dysfunction may be facilitated by mitochondrial dysfunction [29]. Several studies have shown that mitochondrial damage is associated with the progression and severity of MS, and astrocytic mitochondrial dysfunction is correlated with MS progression [70]. As a result of hypoxic injury, pericyte degeneration and capillary construction are exacerbated, thereby inducing further hypoperfusion [27,44,45]. Global hypoperfusion of both white and gray matter is associated with cognitive dysfunction and atrophy in progressive MS [26,27,45].

### 2.7. Therapies Modulating the BBB in MS

Methylprednisolone pulse therapy is widely used for the acute treatment of MS relapses, and it influences the recovery of new Gd-enhanced lesions [71]. Glucocorticoids (GCs) reduce immune cell trafficking and cytokines (IFN-γ, TNF-α, and IL-2) from lymphocytes [72,73]. GCs recover BBB dysfunction through an increase in tight junctions (occludin and claudin), a decrease in MMP-1 and MMP-9 expression, and the downregulation of adhesion molecules, such as VCAM-1, ICAM-1, and E-selectin, in BBB endothelial cells [74,75,76] (Table 1).

IFN-β therapy is the first approved disease-modifying therapy (DMT) that decreases T cell proliferation. IFN-β prevents trans-endothelial migration of proinflammatory CD4+ Th1 cells and enhances BBB integrity through the upregulation of tight junctions [77,78,79].

Natalizumab is a monoclonal antibody against α4β1 integrin, which is the cognate ligand of VCAM-1. This drug cannot cross the BBB but blocks the interaction between α4 integrin from the surface of lymphocytes and VCAM-1 from the surface of BBB endothelial cells, thereby preventing the trans-endothelial migration of lymphocytes directly [80]. Natalizumab is widely used for RRMS patients and has demonstrated high efficacy by reducing the annualized relapse rate and MS lesion accumulation on MRI and by decreasing the sustained progression of disability [81]. Natalizumab dramatically reduces the number of CD4+ T cells, CD8+ T cells, Th17, and B cells in the CNS, as lymphocytes cannot adhere to the BBB after treatment with natalizumab [82]. Unfortunately, the use of natalizumab is associated with a potentially fatal complication in progressive multifocal leukoencephalopathy (PML), as it inhibits immune surveillance against viral leukoencephalopathy induced through infection with John Cunningham (JC) virus [83].

Dimethyl fumarate (DMF) is a first-line oral DMT in RRMS patients, and it has shown efficacy in reducing relapse rates. Activation of the transcription factor pathway nuclear factor (erythroid-derived 2)-like 2 (Nrf2), which maintains intracellular redox homeostasis, is a target of DMF, and activation of the hydroxyl carboxylic acid receptor, independent of the Nrf2 pathway, is another target of DMF [84]. DMF reduces the number of serum lymphocytes, such as CD4+ T cells, CD8+ T cells, B cells, and type 1 myeloid dendritic cells, through the activation of the Nrf2 pathway [85,86]. It also decreases the trans-endothelial migration of lymphocytes by decreasing α4 integrin on the lymphocyte surface and VCAM-1 on the endothelial cell surface independent of the Nrf2 pathway [87]. Furthermore, DMF can cross the BBB and exert a protective effect on neurons and astrocytes by inducing an antioxidant effect dependent on the Nrf2 pathway and modulating microglia independent of the Nrf2 pathway [87,88].

Fingolimod is a sphingosine 1-phosphatate (S1P) receptor modulator that acts on S1P receptors, such as S1P1, S1P2, S1P3, S1P4, and S1P5. Fingolimod reduces the number of lymphocytes in the periphery by inhibiting the egress of lymphocytes from lymph nodes [89,90]. It decreases the trans-endothelial migration of lymphocytes by acting on S1P1 and S1P3 on the surface of BBB endothelial cells [91,92,93]. Fingolimod also modifies the barrier function through clausin-5 upregulation and VCAM-1 downregulation [94]. After fingolimod crosses the BBB, it exerts protective effects on neurons through S1P1 and S1P3 modulation and BDNF upregulation [91,95,96], oligodendrocytes through S1P1 and S1P5 modulation [91,95,97], and astrocytes through S1P1 modulation, and it leads to the inhibition of proinflammatory cytokines and microglia [98,99,100].

Cladribine is a purine nucleoside analog that reduces activated B and CD4+ T lymphocytes [101]. Cladribine can cross the BBB and act on lymphocyte death that has already entered the CNS [102]. Cladribine has an effect on inhibiting lymphocyte trafficking by interacting with ICAM-1 and E-selectin and reducing MMP-2 and MMP-9 secretion [103].

**Table 1:** Effect of DMT on the BBB-ECs.

DMT	Effects on the BBB-ECs	Crosses the BBB?	Reference
Steroid	↑Tight junction (occludin, claudin-5) ↑BBB function ↑TIMP-1 ↓MMP-9	Yes	[72,73,74,75,76]
Interferon-β	↑BBB function ↓VCAM-1, ICAM-1, E selectin ↓Trans-endothelial migration of lymphocyte	Yes, slightly	[72,74,77,78]
Anti-α4 integrin Ab Natalizumab	↓Trans-endothelial migration of lymphocyte (blocks the interaction between α4 integrin and VCAM1)	No	[80]
Anti-CD20 Abs Ofatumumab, Ocrelizumab, Rituximab	Indirect effect due to depletion of B cells ↓Proinflammatory cytokines ↓Complement activity ↓Autoantibody	No	[26,104]
Fingolimod	↓S1P1/S1P3 on the surface of BMECs ↓Trans-endothelial migration of lymphocyte ↓VCAM-1 ↑Tight junction (claudin-5)	Yes	[90,91,92,93,94]
Dimethyl fumarate	↓Trans-endothelial migration of lymphocyte ↓VCAM-1, ICAM-1 ↑BBB function via Nrf2-activity	Yes	[87]
Cladribine	↓ICAM-1, E-selectin ↓MMP-2, MMP-9 ↓CXCL8, CCL5	Yes	[103]
Anti-IL6 Ab Satralizumab	↑BBB function ↓CCL2, CXCL8 ↓Trans-endothelial migration of lymphocyte	No	[105]
Anti-CD19 Ab Inebilizumab	Indirect effect due to depletion of B cells ↓Proinflammatory cytokines ↓Complement activity ↓Autoantibody	No	
Anti-complement Ab Eculizumab Ravulizumab	Unknown	Unknown	

DMT = disease-modifying therapies; BBB-ECs = blood–brain barrier endothelial cells; ↑ = increase; ↓ = decrease; TIMP = tissue inhibitor of metalloproteinases-1; MMP = matrix metalloproteinases; VCAM = vascular cell adhesion molecule; ICAM = intercellular adhesion molecule; CXCL = C-X-C motif chemokine ligand; CCL = chemokine (C-C motif) ligand.

## 3. Neuromyelitis Optica Spectrum Disorder (NMOSD)

### 3.1. Pathophysiological Mechanism Underlying NMOSD

NMOSD is a relapsing neuroinflammatory autoimmune astrocytopathy, and its predominant clinical manifestations are longitudinally extensive transverse myelitis (LETM) and optic neuritis (ON) [106]. Most NMOSD patients have autoantibodies against the water channel aquaporin-4 (AQP4) expressed on astrocyte endfeet; thus, anti-AQP4 antibody detection has been used for the clinical diagnosis of NMOSD patients worldwide [107,108]. Most cases of NMOSD show a relapsing disease course and severe disability without any preventative therapies [109]. Satralizumab, an interleukin-6 receptor (IL-6R) inhibitor, inebilizumab, an antibody against CD19+ B cells, and eculizumab/ravulizumab, an antibody that blocks the C5 component of the complement, were approved for NMOSD therapies after clinical trials [110].

Regarding the pathophysiological mechanism, AQP4-specific B cells (CD19intCD27highCD38highCD180- B cells) and plasmablasts are selectively expanded in the peripheral blood and produce anti-AQP4 antibodies following IL-6 stimulation [111,112]. Serum anti-AQP4 antibodies penetrate the BBB and bind to AQP4 in astrocyte endfeet [113]. Anti-AQP4 antibodies bind to orthogonal arrays of particles (OAPs), which are formed through the aggregation of M-23 AQP4 isoforms [114]. Binding to AQP4 autoantibodies results in AQP loss in astrocytes and deposits of IgG and IgM and complements the rosette pattern around the BBB with cellular infiltrates of neutrophils, eosinophils, macrophages/microglia, and T cells [115,116]. Binding of anti-AQP4 antibodies to AQP4 activates the complement through C1q with anti-AQP4 antibodies, leading to astrocyte death through classical complement cascade activation and membrane attack complex (MAC) formation [115]. C3a and C5a increase vascular permeability and neutrophils [110]. Interactions between pathogenic T cells and B cells in the presence of IL-6, IL-23, and TGF-β differentiate into Th17 cells, which secrete IL-17, promote endothelial activation, and increase trans-endothelial migration of neutrophils and eosinophils [106,117,118]. Regarding the pathological findings of NMOSD, loss of astrocytes, neuronal injury, demyelination, microglial activation, and macrophage infiltration were prominent [116].

### 3.2. Fluid Biomarkers for BBB Disturbance in NMOSD

An increase in Qalb, indicating increased albumin leakage in the CSF, was clinically observed in the acute phase of NMO [119,120]. Well-established fluid biomarkers for predicting the prognosis or treatment response in NMOSD are still insufficient. B cells, neutrophils, and eosinophils infiltrate the CNS across the BBB and contribute to the development of NMOSD lesions [110,121]. Increased levels of B cell activating factor (BAFF), proliferation-inducing ligand (APRIL), IL-6, and CXCL-13, which play a critical role in the survival and homeostasis of B cells, were observed in the CSF of patients with NMOSD, and they probably play an important role in AQP4-antibody-producing cell recruitment and maintenance [122,123]. The number of neutrophils in the CSF is elevated in approximately 60% of acute and untreated NMOSD patients, and neutrophil chemo-attractants CXCL5 and CXCL8 and neutrophil protease are elevated in the sera of NMOSD patients [124,125]. In addition, an increase in eotaxin-2, eotaxin-3, and eosinophil cationic protein (ECP), which contribute to the recruitment and activation of eosinophils, was observed in the CSF of patients with NMOSD compared to patients with multiple sclerosis and healthy controls, and apparent infiltration of eosinophils around the perivascular and meningeal space was observed in the active NMOSD lesion, suggesting the contribution of eosinophils to the pathogenesis of NMOSD [110,123].

IL-6, produced by astrocytes in NMOSD, is associated with increased BBB permeability [126,127]. IL-6 levels in CSF and serum in NMOSD patients are higher than those in MS patients and healthy controls, and they are correlated with the Expanded Disability Status Scale (EDSS) and CSF cell counts [128,129]. The concentration of IL-6 in the serum and CSF is elevated in NMOSD during relapse compared with that in remission, and higher serum concentrations of baseline IL-6 levels in remission are correlated with a higher relapse risk [128,129]. Increased levels of IL-6 in the CSF are linked to short relapse-free durations after relapse [127]. GFAP levels are transiently increased in the CSF and serum during NMOSD attacks and correlate with disability in NMOSD [106,130]. Importantly, serum GFAP levels in AQP4-Ab+ NMOSD during remission may be predictive of future attack risk in NMOSD [131]. Some studies suggest that serum neurofilament light chain L (NfL) may correlate with disability worsening during and after an attack in NMOSD and may serve as an indicator of treatment response in NMOSD [132]. Increased serum GFAP and NfL levels may reflect damage to astrocytes and neurons and the breakdown of the BBB.

Regarding the markers of BBB injury, serum sVCAM-1 and sICAM-1 in NMOSD were increased, but serum sPECAM-1 in NMOSD was decreased compared to that in healthy controls. The concentration of sPECAM-1 was negatively correlated with EDSS in NMOSD patients [133].

### 3.3. Disruption of BBB in NMOSD

When the BBB is disrupted in NMOSD, massive amounts of AQP4 antibodies enter the CNS. Gd-enhanced lesions on MRI and/or increased Qalb were clinically observed in NMOSD during the acute stage [134]. More anti-AQP4 antibodies are produced from serum plasmablasts than from CSF plasmablasts in NMOSD [135,136]. The anti-AQP4 antibodies cannot induce disease development without pre-existing T-cell-mediated CNS inflammation and BBB disruption, as the injection of anti-AQP4 antibodies alone from the periphery is not sufficient to mediate NMO-like histopathology [137,138]. Relatively high titers of anti-AQP4 antibodies are observed in sera from many patients with NMOSD, even during remission, suggesting the importance of BBB disruption in inducing CNS lesions [139,140]. However, the mechanism through which anti-AQP4 antibodies in the sera can bind to AQP4 on astrocyte endfeet behind the BBB in NMOSD has long been unclear [2].

As endothelial cells have weak tight junctions, and the expression of AQP4 is enriched in circumventricular organs (CVOs), including the area postrema, CVOs may be a viable route for the entry of anti-AQP4 antibodies into the CNS [141]. MRI observations show that NMO lesions are often observed in the hypothalamus, the periaqueductal brainstem, and the area postrema surrounding the CVO [142,143]. After AQP4-IgG enters the CSF space through the CVO, it can affect astrocytes via intrathecal inflammation and induce direct damage to the BBB through astrocyte dysfunction [141]. Another possible route is through direct penetration of the BBB. However, serum anti-AQP4 antibodies cannot affect BBB endothelial cells because they do not express AQP4 protein [2]. We hypothesized the presence of other specific autoantibodies against BBB endothelial cells in NMOSD sera that mediate increased penetration of anti-AQP4 antibodies across the BBB [144,145,146,147]. Our data showed that sera from patients with NMOSD during the acute phase decreased the barrier function and claudin-5 protein through VEGF and MMP-2/9 secreted from BBB endothelial cells in an autocrine manner [144,145,146].

**Figure 3:**
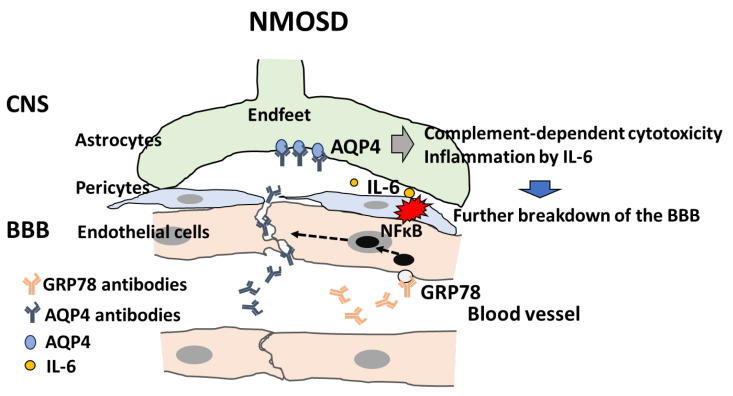
**Possible mechanism of BBB breakdown in NMOSD**. GRP78 autoantibodies in NMO-IgG bind to GRP78 on the blood–brain barrier (BBB) endothelial cells (ECs) and activate nuclear factor-kappa B (NF-κB) signaling in BBB-ECs, leading to enhanced permeability via degradation of tight junctions. Infiltration of AQP4 autoantibodies in NMO-IgG into the CNS causes binding of AQP4 autoantibodies to AQP4 on the endfeet of astrocytes, thus giving rise to complement-dependent astrocyte cytotoxicity. IL-6 secreted by astrocytes induces inflammation (red, collision mark), which further mediates BBB breakdown.

The following results were obtained from our study [147]: (1) IgG from 50 patients with AQP4 antibody-positive NMOSD (NMOSD-IgG) and two monoclonal “not AQP4-specific” antibodies from CSF plasmablasts from NMOSD patients bound to and activated BBB endothelial cells through the NF-κB signal and increased permeability via the decrease of claudin-5 in vitro; (2) glucose-regulated protein 78 (GRP 78) was identified as the antigen of these two monoclonal antibodies; (3) the reduction of GRP78-specific antibodies from NMOSD-IgG decreased the effect on the activation of BBB endothelial cells; and (4) peripheral injection of GRP78-speficic NMO monoclonal antibody induced increased permeability of the BBB in vivo. Our series of studies demonstrated that GRP78 autoantibodies can directly bind to GRP78 on the BBB endothelial cells and mediate the increased permeability of BBB endothelial cells through the activation of NF-κB signaling, thereby causing the paracellular entry of anti-AQP4 antibodies across the BBB endothelial cells [147] (Figure 3). Our study showed that the positivity rate of anti-GRP78 antibodies differed from the NMOSD phenotype (LETM 71% vs. ON 17%), and positivity of anti-GRP78 antibodies in NMOSD was associated with the LETM phenotype and EDSS severity in each patient [148]. GRP78 autoantibodies have been detected in sera of patients with rheumatoid arthritis (RA), and these antibodies are produced in response to abundant GRP78 in the synovial fluids of patients with RA [149].

Another important molecule that plays a critical role in BBB breakdown is IL-6. Some in vitro studies have demonstrated that NMOSD-IgG mediates IL-6 release in astrocytes via JAK/STAT or NF-κB signaling [150,151,152]. Our studies showed that AQP4 Ab-NMOSD IgG-mediated IL-6 production by astrocytes with AQP4 expression and IL-6 signaling to BBB endothelial cells increases barrier permeability, upregulates the expression of chemokines (CCL2 and CXCL8), and reinforces the transmigration of leukocytes under flow according to in vitro static and flow-based BBB models, including co-culture of human brain microvascular endothelial cells (TY10) and human astrocyte cell lines with or without AQP4 expression [152]. Furthermore, satralizumab, an IL-6R-neutralizing antibody, reversed the increased BBB permeability and the infiltration of lymphocytes [105]. Our series of studies showed that the secretion of IL-6 from astrocytes on the CNS side after binding of AQP4 antibodies to AQP4 on astrocytes increased the permeability of the BBB and enhanced the infiltration of inflammatory cells through the upregulation of chemokines (CCL2 and CXCL8) from endothelial cells through IL-6 signaling [105].

## 4. Pathophysiological Mechanism and BBB Breakdown in MOGAD

MOGAD is a recently recognized, new entity in the spectrum of CNS inflammatory demyelinating diseases that differs from both MS and NMOSD [153,154]. The international diagnostic criteria for MOGAD are based on the presence of anti-MOG autoantibodies (MOG-Abs) detected using cell-based assays [153]. The clinical phenotype of MOGAD is broad and includes optic neuritis, transverse myelitis, cerebral cortical encephalitis, brainstem or cerebellar symptoms, and acute disseminated encephalomyelitis (ADEM) [153,154]. MOG is a transmembrane protein on the outer surface of the CNS that is expressed in oligodendrocytes [155,156]. Histopathological findings of MOGAD show a distinct pattern of confluent demyelination around small vessels in white matter and deep gray matter structures with abundant myelin-laden macrophages/microglial cells [157,158,159]. The dominant infiltrating lymphocytes are CD4+ T cells, with few CD8+ T cells and B cells [160,161,162]. A study found that early-phase demyelinating lesions of MOGAD showed MOG-dominant myelin loss with relatively preserved oligodendrocytes [160,161,162].

CSF pleocytosis in MOGAD is common during relapses in the spinal cord (85%) and the brain/brainstem (60%) [163]. Q Alb is increased in almost one-third of patients with MOGAD [164,165]. The cytokines/chemokines in the CSF showed an increase in proinflammatory cytokines/chemokines, including Th1 (TNF-α, IFN-γ), Th2 (IL-13), Th17 (IL-6, IL8, G-CSF, GM-CSF), and B cells (CXCL12, BAFF, APRIL, CXCL13, and CCL19), in MOGAD patients [123,166].

MOG-specific T cells are activated peripherally [167]. Infections, molecular mimicry, and MOG peptide presentation can promote the activation of self-reactive T cells [168]. The pathogenicity of MOG-IgG purified from patients with MOGAD was observed based on the finding that human MOG-IgG was injected intrathecally in an adoptive transfer EAE model induced by myelin basic protein (MBP) or MOG-specific T cells transferred to Lewis rats [169]. In the study, human MOG-IgG was pathogenic in two different EAE models, suggesting that MOG-Abs have a pathogenic effect coupled with MOG-specific or encephalitogenic T cells when they enter the CNS [169]. MOG-Abs cannot bind to or react with the BBB, as MOG is not expressed in BMECs. MOG-Abs are produced mostly peripherally, and these Abs can penetrate the impaired BBB (induced by activated T cells, infection, and co-existing autoantibodies) [170]. Our data demonstrated that MOG-IgG-purified MOGAD patients had their BBB endothelial cells activated during the acute phase, resulting in the induction of NF-κB signaling, increased VCAM-1/ICAM-1, increased permeability, and decreased Nrf2 [170]. The positivity rate of GRP78 autoantibodies in acute MOGAD was 66%, and the removal of GRP78 antibody from MOG-IgG reduced the effect on NF-κB activation, indicating that co-existing anti-GRP78 antibodies with MOG-Ab can facilitate BBB transit of pathogenic MOG-Abs in MOGAD [170].

## 5. NPSLE and AE

### 5.1. NPSLE and the Blood–Brain Barrier

SLE is a multifactorial autoimmune disease with involvement in several areas, including the kidneys, skin, and brain [171]. About 40–75% of patients with SLE experience neuropsychiatric symptoms, termed NPSLE [171,172]. The pathogenesis of NPSLE is considered multifactorial, involving genetic factors, several inflammatory cytokines, autoantibodies, complement activation, and BBB dysfunction [171,172]. The symptoms of NPSLE vary from mild symptoms, such as headache, mood disorder, and cognitive decline, to severe symptoms, such as seizures, cerebrovascular events, an acute confusional state, and psychosis [171,173]. The symptoms of NPSLE are divided into two presentations: focal and diffuse disease. Focal disease observed in stroke and focal seizures is closely associated with antiphospholipid syndrome. Diffuse diseases showing mood disorder, cognitive decline, acute confusion state, and psychosis are related to neurotoxic autoantibodies, cytokine-mediated inflammation, and cell-mediated inflammation [169,173,174]. The diagnosis of NPSLE is difficult because of the lack of accurate and reliable biomarkers. Thus, the diagnosis of NPSLE requires the exclusion of other causes [173,174]. Several diagnostic biomarkers, including serum interleukin (IL)-6, microRNA (miR)-23a, miR-155, and cerebrospinal fluid (CSF) α-Klotho, have been reported to discriminate between patients with NPSLE and controls; however, specific biomarkers that are decisive for the diagnosis are still lacking [175]. Several pathological mechanisms, including neuroinflammation and neuronal damage induced by autoantibodies and proinflammatory cytokines (TNF-α, IL-1, IL-8, and IL-17), vascular occlusion, and BBB breakdown, have been implicated in NPSLE [172,175]. Elevated CSF Qalb and S100B levels in the CSF have been observed in NPSLE, indicating the involvement of dysfunction of the BBB in the development of the disease [176,177].

Vascular pathology is an important factor in NPSLE pathogenesis [171,172]. In patients with focal NPSLE who develop stroke, the direct contribution of the vascular pathology is clear [172]. In contrast, vascular pathology is less obvious in patients with diffuse NPSLE, but pathological findings from autopsied patients have reported microinfarcts, microhemorrhaging, and vasculopathy [172]. Brain MRI also showed large-vessel findings (stroke in large arterial supply territories) and small-vessel disease (lacunar infarcts and microbleeds) [178]. Leukocyte coagulation, immune complex deposition, complement activation, and autoantibody-mediated vascular damage contribute to thrombosis of the large and small vessels in NPSLE [179]. In addition, brain pathology in NPSLE patients revealed cerebral edema, vascular remodeling and wall calcification, diffuse ischemic change, neuronal and myelinated axonal loss, reactive astrocytes, and microglia proliferation, suggesting dysfunction of the BBB and NVU in NPSLE [171,180].

Anti-dsDNA, anti-phospholipid (aPL), anti-ribosomal P protein (anti-P), and NMDA receptor antibodies have been associated with NPSLE manifestations [10,172]. Anti-aPL antibodies can induce vascular endothelial cell injury, platelet activation, and thrombosis, resulting in focal ischemia and intracranial vascular embolism [181,182]. A subset of anti-dsDNA antibodies (anti-dsDNA/NMDAR-NR2 antibodies) can cross-react with NMDAR-NR2 expressed in neurons [10,172,183]. Anti-dsDNA/NMDAR-NR2 antibodies can damage endothelial cells through the upregulation of the NF-κB signal and by secreting inflammatory cytokines, such as IL-6 and IL-8, from endothelial cells [184,185]. After penetrating the BBB, this antibody was shown to be able to induce neuronal apoptosis and the degeneration of surviving neurons in an in vivo model, and it was associated with behavioral and psychiatric manifestations in NPSLE [186,187,188]. Anti-P antibodies can induce apoptosis and dysfunction in hippocampal neurons, causing cognitive impairment after the penetration of the BBB [189].

Anti-endothelial cell antibodies against unknown antigens located on the surface of endothelial cells are common in 65% of NPSLE cases and can contribute to the direct cytotoxic effect induced by complement- or antibody-dependent cytotoxicity and mediate the coagulation of endothelial cells [190,191,192,193]. Diffuse NPSLE with psychosis or depression is associated with serum anti-endothelial cell antibody levels. Anti-Nedd5 antibodies bind to the C-terminal region of Nedd5 and are associated with psychiatric manifestations of NPSLE [194]. In addition, these anti-endothelial cell antibodies from NPSLE patients increased ICAM-1, VCAM-1, and E-selectin expression, as well as the secretion of cytokines, such as IL-1b, Il-8, and MCP-1 [147,191,192]. Anti-GRP78 autoantibodies have been detected in NPSLE, and titers of these antibodies are higher in diffuse NPSLE with acute confusion than in focal NPSLE [195]. A recent study showed that anti-endothelial cell antibodies contribute to the initial stages of vascular damage but not to the development of vasculitis in SLE [192].

Another recent study showed that microglial activation in the brain of SLE model (MRL/lpr) mice and blocking microglial activation through CD40 inhibition improved neuropsychiatric symptoms in mice, suggesting that microglia play an important role in the pathological process in NPSLE [196]. Microglial activation may therefore have an important effect on the disruption of the BBB and NVU in NPSLE.

### 5.2. AE and BBB

AE is associated with autoantibodies against synaptic receptors, neuronal cell surface proteins, and neuronal intracytoplasmic antigens, including NMDAR encephalitis leucine-rich glioma-inactivated 1 (LGI-1), γ-aminobutyric acid type B receptor (GABAbR), and contactin-associated protein-2 (CASPR2) antibodies [197,198,199]. Anti-NMDAR encephalitis was reported to be the most common AE (81%) [200,201]. Young females are often affected, and some develop ovarian teratomas [201]. Ectopic neural tissue in ovarian teratomas as a source of autoantigen is thought to trigger the production of NMDAR autoantibodies in the sera [201]. The symptoms of the disease start with mood changes and psychosis, followed by consciousness disturbance, seizures, respiratory failure, bizarre, involuntary movements, and autonomic disturbances [200]. Anti-NMDAR antibodies are detected in the serum and CSF of patients with NMDAR encephalitis, and anti-NMDAR antibody titers are associated with the severity of disease symptoms, outcomes, and prognosis [200,201]. Anti-NMDAR antibodies can bind to the NMDAR-NR1 subunit and induce the selective internalization of NMDARs, resulting in a decreased glutamate synaptic function [198,202,203,204]. Brain-biopsied or autopsied cases of NMDAR encephalitis showed mild perivascular lymphocytic cuffing, microglial activation, and a decrease in NMDAR expression in the hippocampus [205,206].

An increase in Q-Alb was observed in anti-NMDAR encephalitis, indicating BBB damage [207], which was shown to be associated with the prognosis and mRS score after two months of follow-up; these findings suggest that BBB damage reflects disease severity [207]. Anti-NMDAR antibodies in the sera may penetrate the damaged BBB and enter the CNS, leading to clinical symptoms. NMDAR is expressed on BBB endothelial cells, and the activation of NMDAR can affect the paracellular permeability of the BBB with the altered expression of tight junctions through the activation of the PI3K/Akt signaling pathway [208,209]. Which mechanism at the molecular level is involved in anti-NMDAR encephalitis remains unclear, as does whether or not BBB dysfunction occurs in other types of AE with anti-LGI-1, anti-CASPR2, or anti-GABAbR autoantibodies. Further analyses are needed to understand the molecular mechanisms responsible for BBB breakdown in AE.

Autoimmune cerebellar ataxia is an emerging disease that affects the cerebellum via autoimmune mechanisms [210,211]. The disease has several etiologies, including gluten ataxia, anti-glutamate decarboxylase (GAD) ataxia, paraneoplastic cerebellar degeneration (PCD), primary autoimmune cerebellar ataxia, and postinfectious cerebellar ataxia [210,211]. Breakdown of the BBB could potentially explain the vulnerability of the cerebellum to autoimmune cerebellar ataxia, as it triggers the entry of pathogenic autoantibodies or lymphocytes induced by the autoimmune response in the periphery into the cerebellum.

Whether or not BBB permeability is increased in autoimmune cerebellar ataxia remains unclear.

## 6. Paraneoplastic Neurological Syndromes (PNSs)

### 6.1. PNSs and the BBB

PNSs are characterized by acute or subacute neurological manifestations and mediated by the remote effects of cancer, with an immune-mediated pathogenesis that is not caused by cancer or metastasis [212,213]. Recent diagnostic criteria have demonstrated that high-risk phenotypes of PNS include encephalomyelitis, limbic encephalitis, rapidly progressive cerebellar syndrome, opsoclonus myoclonus, sensory neuronopathy, gastrointestinal pseudo-obstruction (enteric neuropathy), and Lambert–Eaton myasthenic syndrome (LEMS) [214]. Anti-onconeural antibodies directed to intracellular antigens (Hu, Yo, Ri, MA1/2, CRMP5), intracellular synaptic antigens (GAD65, amphyiphysin), and extracellular/cell membrane antigens (NMDAR, AMPAR, LGI1, CASPR2, GABABR, mGluR1, GlyR, VGCC, mGluR5) have been identified as being associated with PNSs and are thus used for their diagnosis [214,215,216].

Paraneoplastic cerebellar degeneration (PCD) is one of the most common forms of PNSs [212,217]. Approximately half of PCD cases are related to anti-Yo antibodies and other autoantibodies, including anti-Hu, anti-Ri, anti-Tr, anti-Ma2, anti-P/Q-type VGCC, and anti-CV2/CRMP5 antibodies [212,218,219]. Dysfunction of the BBB or blood–nerve barrier (BNB) may be responsible for the onset and progression of PNSs, although the precise molecular mechanism underlying the BBB breakdown in PNSs remains elusive.

### 6.2. PCD-LEMS

LEMS is an autoimmune disease of the neuromuscular junction characterized by proximal muscle weakness, areflexia, and autonomic dysfunction, and it is associated with P/Q-type VGCC autoantibodies and small-cell lung carcinoma [216,220,221]. P/Q-type VGCCs are localized at presynaptic motor nerve terminals and play a role in neurotransmitter release [220,221]. Cerebellar symptoms are observed in 10% of LEMS patients diagnosed with PCD with LEMS (PCD-LEMS) [216]. In autopsied brains of PCD-LEMS patients, the selective reduction of P/Q-type VGCCs was observed in the molecular layer of the cerebellum. Anti-P/Q-type antibodies can enter the CNS in cases of BBB dysfunction in PCD-LEMS [222].

**Figure 4:**
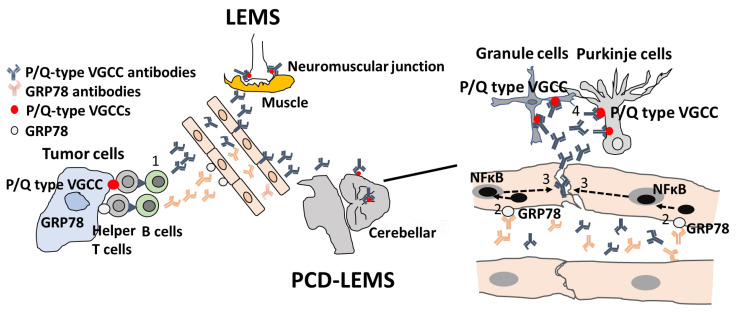
**Possible mechanism of BBB breakdown in PCD-LEMS.** Glucose-regulated protein (GRP) 78 autoantibodies and P/Q-type voltage-gated calcium channel (VGCC) autoantibodies are produced through cross-reactivity with tumor cells, as both GRP78 and P/Q-type VGCC are expressed on the surface of tumor cells (1). After GRP78 autoantibodies bind to GRP78 on endothelial cells, the permeability of the BBB is increased via the activation of nuclear factor-kappa B (NF-κB) (2), thereby inducing the infiltration of P/Q-type VGCC autoantibodies into the CNS space (3). Binding of P/Q-type VGCC autoantibodies to P/Q-type VGCC causes injury to Purkinje and granule cells (4).

We recently identified anti-GRP78 antibodies in patients with PCD-LEMS and NMOSD [223]. GRP78 (heat shock protein family A [Hsp70] member 5 HSPA5) plays a role in preventing unfolded protein accumulation and apoptosis as an endoplasmic reticulum (ER) chaperone in all CNS cells [224]. The cell surface GRP78 is abundant in malignant cells and BBB endothelial cells in vivo and in vitro, leading to the activation of NF-κB signal transduction, which supports the notion that cell-surface GRP78 may be a target for cancer-specific therapy [225,226,227]. GRP78 autoantibodies have been detected in sera from patients with malignant tumors, suggesting that GRP78 autoantibodies may be produced in response to cell-surface overexpression of GRP78 in patients with malignant tumors [228,229,230]. In PCD-LEMS, GRP78 antibodies induced by cross-reactivity with small-cell carcinoma can induce BBB dysfunction and facilitate the penetration of anti-P/Q-type VGCC antibodies into the cerebellum, resulting in cerebellar ataxia [223] (Figure 4).

### 6.3. Paraneoplastic NMOSD

In addition, cases of paraneoplastic NMOSD have been reported increasingly frequently, and some case reports of patients with paraneoplastic NMOSD have shown the expression of AQP4 in the tumor cells, suggesting that AQP4 autoantibodies may be produced in the autoimmune response to AQP4 protein in the tumor cells of some paraneoplastic NMOSD patients [231,232,233]. Our report describes a case of paraneoplastic NMOSD presenting with LETM with colorectal cancer that was positive for both GRP78 antibodies and AQP4 antibodies [234]. In that case, the tumor cells showed a high expression of GRP78, which possibly upregulated the production of GRP78 antibodies because of an autoimmune response mediated by the tumor.

## 7. Conclusions

In this review, we summarize the pathogenic contribution of BBB alteration in several neuroimmunological diseases, such as MS, NMOSD, MOGAD, AE, and PNSs. The major molecular mechanisms responsible for BBB dysfunction differ among diseases; thus, a detailed understanding of the pathomechanism involved needs to be explored in greater depth to stimulate drug development to improve BBB function and treatment options. Common genetic and environmental factors, including dietary habits, Vitamin D, the gut microbiome, smoking, and EBV infection, may contribute to BBB dysfunction in several neuroimmunological diseases, including MS. Although, at present, several DMTs, such as natalizumab for MS and satralizumab for NMOSD, can modify BBB function and contribute to relapse prevention, prospective therapeutic approaches, such as monoclonal antibodies targeting another CAM, cytokines, or chemokines, may pave the way for new treatments to prevent BBB injury in several neuroimmunological diseases.

Furthermore, investigation of how the BBB is repaired in neuroimmunological disease and how neuroprotective medicines penetrate the BBB may lead to the discovery of novel molecular-targeted drugs against the BBB for several neurological diseases. In particular, as dysfunction of the NVU is associated with gray matter atrophy in MS, several platforms, such as organ-on-a-chip models, have been established in the field of the BBB to understand the detailed pathomechanism of neurological disease, drug discovery research, and screening, leading to further novel therapeutic approaches in several neuroimmunological diseases [4,235,236,237]. Thus, the BBB may become a therapeutic target in several neuroimmunological diseases not only to protect and repair the BBB when damaged but also to reach neuroprotective molecules inside of the CNS.
